# The Effect of a Green Smoothie on Microhardness, Profile Roughness and Color Change of Dental Restorative Materials

**DOI:** 10.3390/polym14102067

**Published:** 2022-05-19

**Authors:** Nikolina Nika Veček, Matej Par, Eva Klarić Sever, Ivana Miletić, Silvana Jukić Krmek

**Affiliations:** 1 School of Medicine, University of Split, Šoltanska 2, 21000 Split, Croatia; veceknika@gmail.com; 2 Department of Endodontics and Restorative Dentistry, School of Dental Medicine, University of Zagreb, Gunduliceva 5, 10000 Zagreb, Croatia; eklaric@sfzg.hr (E.K.S.); miletic@sfzg.hr (I.M.); jukic@sfzg.hr (S.J.K.)

**Keywords:** green smoothie, resin composites, alkasite, glass hybrid, microhardness, roughness, color

## Abstract

Acidic drinks are known to exert negative effects on the surface properties of dental restorative materials. However, the effect of increasingly popular green smoothie drinks has not been addressed so far. The present study investigated the effect of cyclic immersions (5 min daily over 30 days) in a green smoothie drink on the surface properties of contemporary dental restorative materials, including resin composites, an alkasite, and a glass hybrid. Vickers microhardness, profile roughness, and perceptible color change in the CIE L* a* b* color space were evaluated as clinically relevant properties of the material surface. After 30-day green smoothie immersion, microhardness values either decreased by 8–28% (for resin composites) or increased by up to 91% (for glass hybrid). The increase in profile roughness (Ra parameter) of smoothie-immersed specimens was 7–26 times higher compared to the control group. The perceptible color change (ΔE*) in the smoothie group was 3–8 times higher compared to the control group. Overall, this study demonstrated that daily exposure of dental restorations made from resin composites, alkasites, and glass hybrid materials to a green smoothie drink can significantly accelerate material degradation, which is reflected as surface softening, as well as higher roughness and higher perceptible color change.

## 1. Introduction

Owing to the accelerating development and progress of dental medicine in the field of restorative dentistry, a wide range of different materials is available to dental practitioners [1,2]. Polymeric resin composites are the dominant group among contemporary dental restorative materials which fulfill most of the mechanical, chemical, and esthetic requirements for permanent restorations [3,4]. Resin composites are mainly composed of methacrylate matrix and reinforcing glass fillers; however, various modifications of their basic formulation have been introduced and successfully used in clinical practice [5]. A recently launched restorative material termed “alkasite” can be envisioned as a modified resin composite that is capable of releasing fluoride, calcium, and hydroxyl ions, resulting in a potential anti-cariogenic activity [6,7]. Another major group of dental restorative materials includes glass hybrids, which in their latest iteration have sufficiently improved mechanical properties and wear resistance for being used in stress-bearing areas [8].

Regardless of their various composition and properties, the common challenge faced by all dental restorative materials is permanent exposure to the complex environment of the oral cavity, featuring significant changes in temperature, acidity, humidity, and different chemical compounds due to the intake of food and drinks [9]. These conditions cause a gradual degradation of mechanical and chemical properties, consequently affecting the service life of dental restorations [10]. A number of previous studies have shown that acidic dietary foods and beverages can cause surface degradation for various types of dental restorative materials [11,12,13,14,15,16,17,18,19,20]. Concurrently with the increasing consumption of soft and fruit drinks, their potential for damaging dental hard tissues and restorative materials is becoming an important factor affecting the lifespan of contemporary dental restorations [21]. Among various soft and fruit drinks, smoothie drinks are becoming increasingly popular, as exemplified by the fact that the consumption of shop-purchased smoothie drinks in the United Kingdom has risen from 6 million liters in the year 2001 to 51 million liters in the year 2010 [22]. The consumption of smoothie drinks cannot be precisely quantified, as the reported figures exclude consumption of homemade drinks, as well as those bought from cafes, coffee shops, and juice bars. A particular subgroup among smoothie drinks includes the so-called green smoothies, which are especially popular among individuals who prefer consuming raw food [23]. These drinks are commonly composed of blended leafy vegetables, fruit, nuts, and seeds, in various homemade or commercially available recipes [24].

As the influence of green smoothie drinks on surface properties of dental materials has not been examined so far, the present study aimed to evaluate the effect of a 30-day cyclic exposure to a green smoothie made of apple, pear, avocado, and spinach on clinically relevant surface properties (microhardness, profile roughness, and color change) of representative contemporary restorative materials. The first null hypothesis assumed no differences in the tested properties between restorative material specimens that were either immersed continuously in distilled water (control group) or exposed for 5 min daily over 30 days to a green smoothie drink (experimental group). The second null hypothesis assumed that the extent of change of the tested properties would be the same among the materials within each group.

## 2. Materials and Methods

### 2.1. Specimen Preparation

Five contemporary dental restorative materials were investigated, including resin composites (two conventional composites, one bulk-fill composite, and one “alkasite”) and a glass hybrid restorative material (uncoated and coated with a resinous coat). All materials were of A2 shade. The composition of tested materials as provided by their respective manufacturers and the available literature [25,26,27,28] is shown in Table 1.

**Figure 1:**
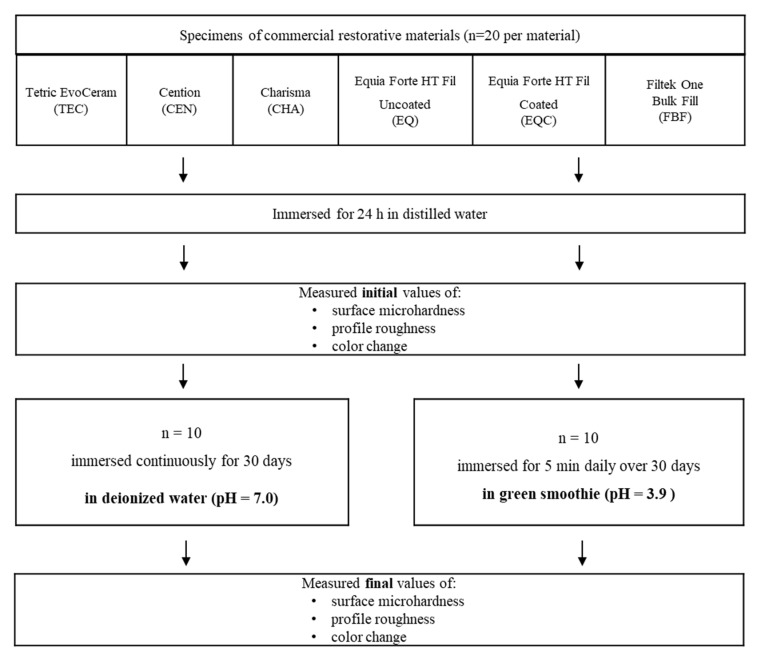
Flowchart of the experimental protocol.

A schematic representation of the study protocol is shown in Figure 1. Twenty specimens were prepared for each experimental group by casting unset materials into discoid Teflon molds (15 mm in diameter and 1 mm in height) and covering them from both sides with a polyethylene terephthalate film. The resin composite specimens were light-cured using a LED curing light (Bluephase G2; Ivoclar Vivadent, Schaan, Liechtenstein) for 20 s with a radiant exitance of 1200 mW/cm^2^, was measured using a calibrated and National Institute of Standards and Technology (NIST)-referenced ultraviolet-visible (UV–Vis) spectrophotometer system (MARC; BlueLight Analytics, Halifax, Canada). All of the measurements described below (microhardness, profile roughness, color change) were performed on the irradiated side of the light-cured specimens. The glass hybrid specimens were left undisturbed to set for 10 min. For the EQC group, a filled resin coating material (Equia Coat, GC, Tokyo, Japan) was applied to all specimen surfaces and light-cured for 20 s from all sides.

All specimens were stored individually in closed containers in 5 mL of distilled water in a laboratory incubator at 37 °C for 24 h. Half of the specimens for each material (*n* = 10) were immersed over 30 days for 5 min daily in 5 mL of commercially available smoothie drink “Green avocado” (Ortoromi, Borgoricco, Italy) containing apple juice (47%), pear juice (25%), avocado (20%), spinach (8%), and ascorbic acid. After the 5-min immersion in the green smoothie at 37 °C, the specimens were re-immersed in a fresh 5 mL of distilled water and stored in the incubator until the next day. As preliminary measurements indicated that pH changes of the green smoothie during the 5-min immersion of restorative material specimens were below 0.2 pH units, pH monitoring was not performed. The other half of the specimens (*n* = 10) represented the negative control group and were immersed for 30 days in 5 mL of distilled water, which was replaced daily.

### 2.2. Surface Microhardness Measurements

Vickers microhardness (MH) of the material surface was determined with a microhardness tester (CSV-10; ESI Prüftechnik GmbH, Wendlingen, Germany) using a load of 100 g and a dwell time of 10 s. MH was evaluated at two time points: 24 h after specimen preparation, and after 30 days of immersion. Five MH readings were made for each specimen, and the mean value of these readings was used as a statistical unit in the analysis [7].

### 2.3. Profile Roughness Measurements

Profile roughness parameter Ra was tested using a portable profile roughness tester (Surftest SJ-210; Mitutoyo, Houston, TX, USA). Profile roughness was measured at two time points: 24 h after specimen preparation, and after 30 days of immersion. The following measurement parameters were used: stylus speed: 0.1 mm/s, stylus force: 4 mN, cut-off length: 0.25 mm, sampling length: 0.8 mm, number of sampling lengths: 5. Evaluations were done at three different sites of each specimen within a radius of 3 mm from the specimen center and the mean value was calculated.

### 2.4. Color Measurements

Color measurements were performed using a standardized white background and a probe spectrophotometer (VITA Easyshade III, VITA, Bad Säckingen, Germany). Color measurements were performed at two time points: 24 h after specimen preparation, and after 30 days of immersion. For each specimen, three measurements were taken, and mean values for L*, a*, and b* were recorded to calculate the perceptible color difference (ΔE*) between the two measurement time points.

### 2.5. Statistical Analysis

Normality was tested using the Shapiro–Wilk test and inspection of normal Q–Q plots. Homogeneity of variances was verified by Levene’s test. Changes in MH and Ra between initial and final values were compared using a paired t-test. Independent observations t-test was used for ΔMH, ΔRa, ΔE*, ΔL*, Δa*, and Δb* to compare the extent of change between the smoothie and the control group. For the aforementioned delta variables, one-way ANOVA followed by Tukey post hoc tests were used for comparisons among the materials. To explore correlations among the delta variables, i.e., the combinations of 3 parameters (delta MH, delta Ra, and delta E) × 2 immersion protocols (smoothie and control), a principal component analysis with varimax rotation and Pearson’s correlation analysis were used. All analyses were performed at an overall significance level of 0.05, using SPSS 25 (IBM, Armonk, NY, USA).

## 3. Results

**Figure 2:**
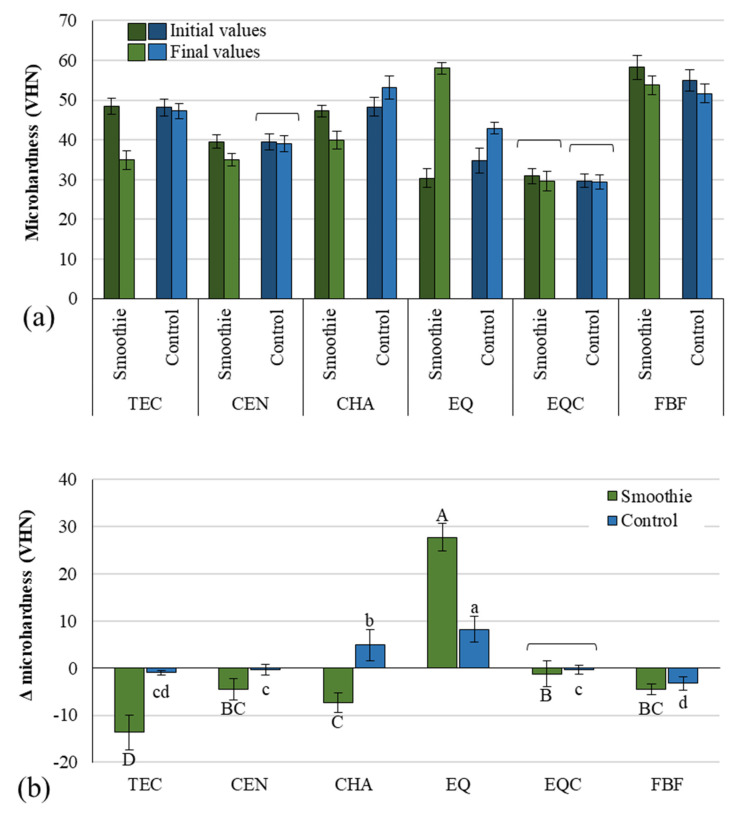
Microhardness (mean values ± 1 standard deviation, *n* = 10) measured 24 h after specimen preparation and after 1 month of immersion (**a**), and delta microhardness (mean values ± s.d.) representing microhardness change between these two time points (**b**). Square brackets indicate statistically similar values. Same uppercase letters indicate statistically similar values within the smoothie group. Same lowercase letters indicate statistically similar values within the control group. TEC: Tetric EvoCeram, CEN: Cention, CHA: Charisma Classic, EQ: Equia Forte HT Fil Uncoated, EQC: Equia Forte HT Fil Coated FBF: Filtek Bulk Fill.

MH data before and after immersion are presented in Figure 2a. A statistically significant MH decrease after immersion was identified for TEC and FBF in both groups. In contrast, for EQ, the MH increased significantly after immersion in both groups, while no significant MH change was identified for EQC (both groups) and CEN (control group). For CHA, MH decreased in the smoothie group but increased in the control group. The comparison of ΔMH between the smoothie and the control group (Figure 2b) shows that the differences were statistically significant for all materials, except for EQC.

**Figure 3:**
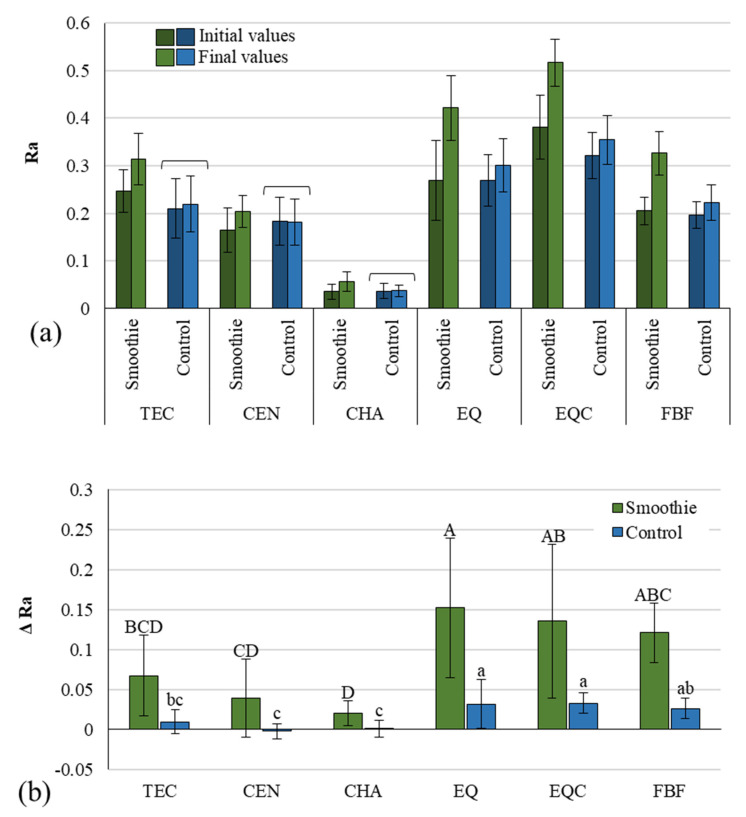
Profile roughness represented as Ra (mean values ± 1 standard deviation, *n* = 10) measured 24 h after specimen preparation and after 1 month of immersion (**a**), and delta Ra (mean values ± s.d.) representing profile roughness change between these two time points (**b**). Square brackets indicate statistically similar values. All pairwise comparisons (smoothie vs. control) of delta Ra values showed statistically significant differences. Same uppercase letters indicate statistically similar values within the smoothie group. Same lowercase letters indicate statistically similar values within the control group. TEC: Tetric EvoCeram, CEN: Cention, CHA: Charisma Classic, EQ: Equia Forte HT Fil Uncoated, EQC: Equia Forte HT Fil Coated FBF: Filtek Bulk Fill.

Profile roughness parameter Ra before and after immersion is presented in Figure 3a. For most experimental groups, Ra values increased significantly after immersion, except for the control groups of three materials (TEC, CEN, and CHA), for which no statistically significant changes after immersion were identified. The comparison of delta Ra between the smoothie and the control group (Figure 3b) shows significantly higher changes of Ra in the smoothie groups compared to the control groups for all materials.

**Figure 4:**
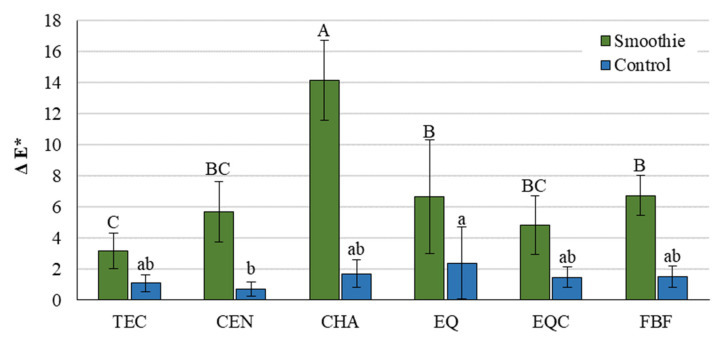
Delta E (mean values ± 1 standard deviation, *n* = 10) representing perceptible color change between 24 h and 1 month. All pairwise comparisons (smoothie vs. control) showed statistically significant differences. Same uppercase letters indicate statistically similar values within the smoothie group. Same lowercase letters indicate statistically similar values within the control group. TEC: Tetric EvoCeram, CEN: Cention, CHA: Charisma Classic, EQ: Equia Forte HT Fil Uncoated, EQC: Equia Forte HT Fil Coated FBF: Filtek Bulk Fill.

Color change expressed as delta E is represented in Figure 4. The extent of color change varied among the materials and was significantly higher in the smoothie group than in the control group for all the materials.

**Figure 5:**
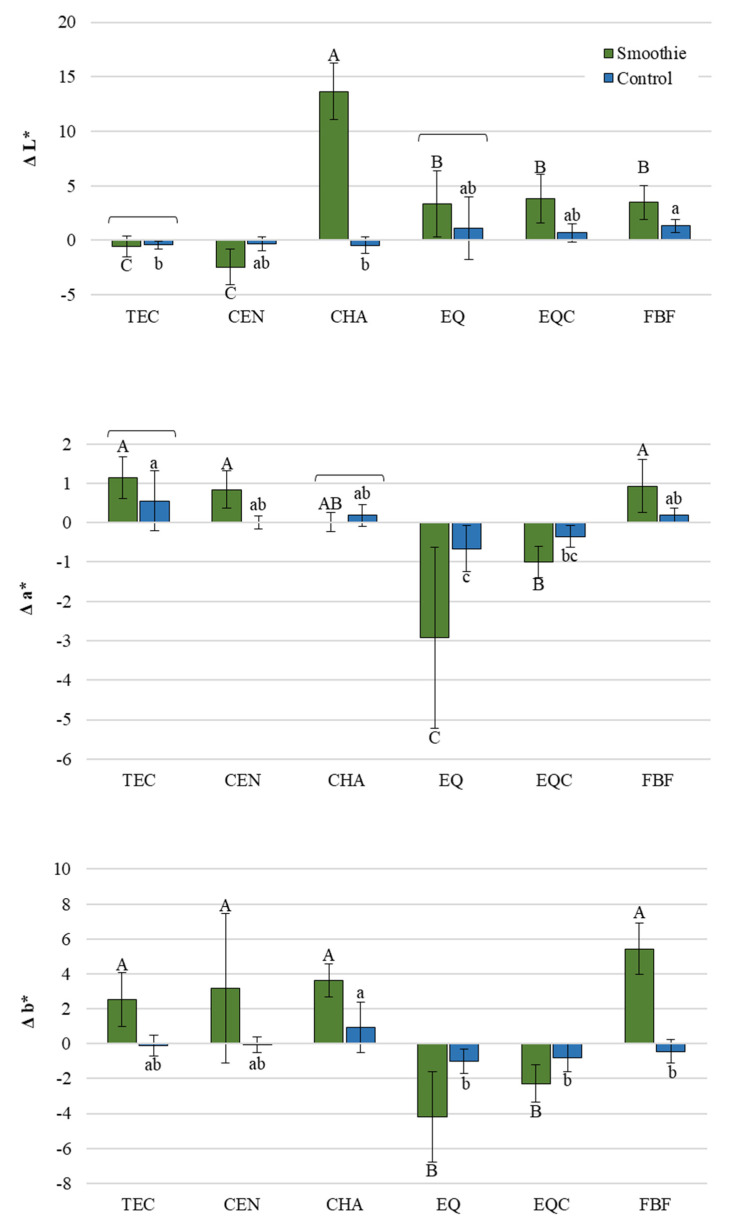
ΔL*, Δa*, and Δb* (mean values ± 1 standard deviation, *n* = 10) representing shifts along individual color axes between 24 h and 1 month. Square brackets indicate statistically similar values. Same uppercase letters indicate statistically similar values within the smoothie group. Same lowercase letters indicate statistically similar values within the control group. TEC: Tetric EvoCeram, CEN: Cention, CHA: Charisma Classic, EQ: Equia Forte HT Fil Uncoated, EQC: Equia Forte HT Fil Coated FBF: Filtek Bulk Fill.

Figure 5 shows the shifts along individual color axes (ΔL*, Δa*, and Δb*). The color changes generally occurred in the same direction in both the smoothie and the control group, with the extent of change being significantly higher in the smoothie group. Several exceptions were observed, i.e., for some materials, the changes were statistically similar for both groups (TEC and EQ for ΔL*; TEC and CHA for Δa*), while for some materials the direction of change differed among the groups (positive vs. negative changes in ΔL* for CHA, and Δb* for FBF).

**Figure 6:**
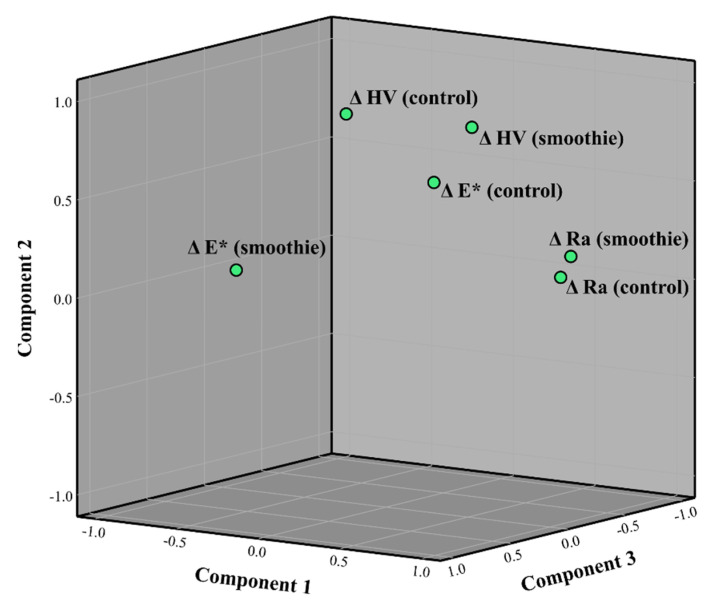
The component loading plot in rotated space for the variables representing the change of microhardness (ΔMH), profile roughness (ΔRa), and perceptible color (ΔE*) within the smoothie and the control group.

The loading plot of the first three principal components in rotated space (Figure 6) indicates relative amounts of covariance among ΔE*, ΔMH, and ΔRa in the smoothie and the control group. The spatial relationship of the variables ΔRa (control) and ΔRa (smoothie) in the rotated space indicates a high amount of joint variability in comparison to the other variable combinations. This was confirmed by the results of a Pearson’s correlation analysis, which showed that for the aforementioned six pairs of variables, bivariate correlations were statistically significant only for the combination of ΔRa (control) and ΔRa (smoothie), with *p* < 0.001 and R = 0.98.

## 4. Discussion

This study investigated the effect of cyclic immersions (5 min daily over 30 days) in a green smoothie drink on the surface properties of restorative materials. While profile roughness and perceptible color change increased after 30 days for all of the investigated materials regardless of the immersion medium, microhardness changes after 30 days occurred in both directions, notably depending on the material and immersion medium. Both null hypotheses were thus rejected.

Unlike profile roughness and perceptible color change, MH was the only parameter in this study that showed bidirectional changes with aging. This behavior occurred because MH changes resulted from two concurrently occurring processes that affect the material’s micromechanical properties in opposing directions: (I) the slow continuation of the setting reaction that has been reported to occur for up to one month in composites [29] and even one year in glass hybrid cement [30], and (II) the gradual degradation of material structure caused by water uptake from the surrounding solution [31]. Our results reflect the outcomes of the competition between these two processes. This was especially evident for resin composites in the control group, which showed highly material-dependent behaviors: for FBF, the plasticization/softening processes dominated and led to an overall MH decrease, for TEC and CEN the degradation was mostly offset by the post-cure polymerization, leading to practically no MH change, whereas for CHA the degradation processes were surpassed by the post-cure polymerization, leading to an overall MH increase. In contrast to these mixed outcomes in the control group, in the smoothie group degradation processes dominated for all resin composites, resulting in material softening. It should be noted that the relative extents of MH decrease for the resin composites in the control group differed from those in the smoothie group, indicating that material degradation in a pH-neutral environment was not related to the degradation extent caused by a cyclic exposure to the green smoothie. The most notable example of this fact was observed for CHA, which showed the highest MH increase after water immersion among all tested resin composites, which was strongly contrasted with the same material demonstrating the second-highest MH decrease when immersed in the smoothie. This consideration is in agreement with no correlation of ΔMH between the control and smoothie group, indicating that the extent of material softening in one immersion medium does not indicate its susceptibility for softening in another medium. Overall, the highest extent of softening due to smoothie immersion was identified for TEC which may have been due to the interface of its pre-polymerized fillers and polymer matrix being prone to degradation. Interestingly, a modest MH decline was observed for CEN compared to the MH values observed for other resin composites. As CEN contains reactive glasses that have been reported to release high amounts of calcium, phosphate, and fluoride ions under acidic conditions [32], it was surprising to observe that its surface resisted the smoothie-induced softening statistically similar to or even better than some of the conventional non-releasing composites that contain only inert fillers (TEC and CHA).

In contrast to resin composites, glass ionomers are not softened by water immersion but rather undergo a maturation process, i.e., the continuation of the setting reaction which gradually improves the material’s mechanical properties [33]. The finding of EQ showing an increase in MH after 30 days of immersion in distilled water was therefore expected. For this material, the MH increase amounted to 24% of its initial value, resulting in a significantly higher increase among all of the materials in the control group. There was, however, an unexpected finding of an even more pronounced MH improvement amounting to 91% for EQ after 30-day smoothie immersion. Although the acidic medium was expected to erode the surface and decrease its MH [34], a previous study has shown that the surface properties of glass ionomers can vary considerably depending on the environment in which the maturation process takes place, for example, 30-day maturation of a conventional glass ionomer cement in saline improved its MH for 50% and 25% in Coca-Cola, while an MH decrease was observed in orange juice (7%) and apple juice (50%) [30]. Hence, the MH changes of glass ionomers immersed in acidic drinks appear to be capable of change in either direction, depending on material and immersion medium characteristics.

In contrast to the uncoated glass hybrid specimens (EQ), which for both the control and the smoothie group showed the significantly highest MH increase after immersion, the coated glass hybrid specimens (EQC) showed no significant MH changes after 30 days, with no significant differences between the control and the smoothie group. This was the result of the resinous coat being approximately three times thicker than the indentation depth of the Vickers pyramid, as identified by the light microscopic measurements of indentation dimensions and coating layer thickness performed as part of MH measurements. Hence, the MH data measured for the EQC group essentially reflected the micromechanical properties of the coating resin instead of the underlying glass hybrid material.

Overall, our MH results show that after 30-day green smoothie immersion, MH values either decreased by 8–28% (for resin composites) or increased by 91% (for glass hybrid). While the MH increase in the glass ionomer was higher than a previously reported increase of up to 50% for a similar material immersed in acidic drinks [30], the data for resin composites were within the range of MH decrease reported by other studies. Namely, the study by Borges et al. reported that 30-day cyclic immersion in acidic drinks diminished MH of composites to the following extents: juice 3–18%, red wine 4–20%, and Coca-Cola 9–22% [13]. Another study showed that Coca-Cola immersion for 15 days decreased MH of resin composites and compomers by 13–22% [14]. Additionally, 1-month immersion in multivitamin syrups and effervescent tablets was shown to diminish MH values by 21–28% for a resin composite, and 13–35% for a glass hybrid [15].

The roughening of the restorative material surface is an unavoidable consequence of its exposure to water and other liquids during the restoration’s service life [35]. The extent of roughening is known to depend both on material characteristics and environmental conditions [36]. Our results indicate that some of the tested materials maintained their surface polish (TEC, CEN, and CHA) under the conditions of neutral pH, while the other materials (EQ, EQC, and FBF) showed a significant increase in roughness despite being immersed in a non-aggressive medium. In contrast, the erosive potential of the green smoothie drink led to a significant Ra increase for all materials, with ΔRa values 7–26 times higher compared to the control group. This wide range of values is indicative of a highly material-dependent behavior and is above the range of results reported in the literature, namely ΔRa for glass ionomers and composites immersed in energy drinks and Coca-Cola was 2–13 times higher than in the distilled water [16], while for multivitamin syrups and effervescent tablets, 2–4 times higher ΔRa values were reported [15]. Although the results of these studies cannot be directly compared to our data due to different experimental conditions, the considerable erosive potential of a green smoothie indicated by the present study should be further investigated.

Regardless of the immersion medium, the glass hybrid groups EQ and EQC showed the significantly highest ΔRa among all of the tested materials. This increase in profile roughness of glass ionomers occurs as a side-effect of their surface dissolution which regularly occurs in this material class and is responsible for ion release [37]. The dissolution of glass ionomers is faster under low pH conditions [38], which explains the ΔRa values in the smoothie group for EQ and EQC 4–5 times higher compared to the control group. Statistically similar ΔRa values for coated and uncoated glass ionomer specimens observed in both immersion media suggest that the resinous coat did not affect profile roughening.

It is interesting to note that ΔRa values for the ion-releasing material CEN were at the low-end among all tested materials; after distilled water immersion no significant increase in its profile roughness was identified, while after smoothie immersion its roughness increased to the extent which was statistically similar to that of the non-releasing composites TEC and CHA. Additionally, Cention immersed in the smoothie showed ΔRa values significantly lower than the glass hybrid and FBF. Considered together with the favorable MH results, the Ra results for CEN indicate that its ion-releasing capability [32,39] does not affect surface degradation any more than was measured for the non-releasing composites TEC, CHA, and FBF, at least in the present in vitro experiment and short-term measurement period of 30 days.

ΔE* values indicate that color change occurred in all materials regardless of the immersion medium, whereas the smoothie group showed 3–8 times higher ΔE* than the control group. Δ E* values in the control group ranged between 0.7–2.4 and were most statistically similar among the materials. Only the ΔE* values at the high-end of this range can be regarded as barely perceptible to the human eye as ΔE* = 2 represents the limit of perceptibility for the untrained observer [40]. Another commonly used threshold used for evaluating the clinical acceptability of color change in dental restorative materials is set at ΔE* = 3.3 [41,42]. This limit was surpassed in the smoothie group by most of the materials, which showed ΔE* in the range of 3.2–14.2, indicating much more noticeable color differences. Except for the very high ΔE* values for CHA (14.2), the ΔE* values of all other materials after smoothie immersion were within the ΔE* range of 3.2–6.7, which is in accordance with previously reported ΔE* values, as follows: 0.7–4.9 for resin composites and compomers after immersion in coffee, orange juice, energy drink, and Coca-Cola [17], 2.1–7.7 for resin composites in coffee, tea, and red wine [18]; 2.3–6.3 for resin composites in coffee, tea, red wine, and Coca-Cola [19]; and 1.8–6.7 for a composite in a strong alcoholic drink, red wine, and soft drink [20].

The color change of restorative materials is the result of the joint action of material degradation and surface staining by the uptake of pigmented compounds from the immersion medium [43]. In the control group, staining was exclusively due to material degradation, whereas in the smoothie group both processes acted synergistically to produce more extensive staining. To explore the individual color changes, in addition to the overall color change represented by ΔE*, each component of the L*a*b* color space was evaluated separately. The lack of a particular pattern in color shifts along a certain color axis indicates that the color change was a result of material-specific degradation processes instead of being a simple uptake of pigments from the smoothie drink. If the extrinsic staining was the primary mechanism of discoloration, more consistent negative changes along the a* axis (shift towards green) and positive changes along the b* axis (shift towards yellow) would be expected to occur for all materials, as green and yellow were the dominant colors of the green smoothie drink. Since no such consistency was observed, it can be inferred that the color change was dominantly due to accelerated degradation which affected the color shifts along the L*, a*, and b* axes for each material differently.

As all of the surface properties evaluated in this study reflect the underlying degradation of the material structure due to immersion in either a neutral or acidic medium, it was hypothesized that some of the measured parameters (ΔMH, ΔRa, and ΔE*) may be correlated. The principal component analysis and Pearson’s correlation analysis showed that only ΔRa (smoothie) and ΔRa (control) were significantly correlated, while no other parameter combinations showed any significant correlations. The practical implication of this finding is that the materials that roughened the most under the neutral pH will also roughen to the highest extent when immersed in the smoothie drink (and vice versa). The lack of analogous correlations between the control and the smoothie group for the other two parameters (ΔMH and ΔE*) show that for these properties the changes under the neutral pH group did not occur commensurably to the changes caused by the smoothie immersion. In addition, the lack of correlations among other binary combinations of ΔMH, ΔRa, and ΔE* indicates that surface softening, roughening, and staining was material-dependent to such an extent that all of the variables changed independently of each other, i.e., that the observed changes in individual properties could not be reduced to an underlying fundamental degradation process. On the contrary, all of the investigated materials underwent considerably different degradation processes that affected individual surface properties to a different degree. Hence, the changes in surface properties for the tested materials cannot be generalized.

Similar to all studies on commercial dental materials, the present study is disadvantaged by investigating a heterogeneous set of complex materials, whose compositions are proprietary to their respective manufacturers and hence only partially disclosed [44]. The commercial dental materials are fine-tuned to fit a particular indication, yet, such systems can only be investigated as a complete product, without being able to systematically evaluate the contributions of individual material components. Hence, no detailed composition/structure/property relationships can be studied as only the overall, integrated, behavior of the whole material is measured [45]. Despite this limitation, evaluating the material behavior is clinically relevant because dental restorative materials are used by practitioners as finished products with compositions being pre-defined by manufacturers. Dental practitioners choose from a variety of available products but unavoidably observe their behavior in a “black box” manner, without being able to understand or adjust the contributions of individual material components. discuss the results and how they can be interpreted from the perspective of previous studies and of the working hypotheses. The findings and their implications should be discussed in the broadest context possible. Future research directions may also be highlighted.

## 5. Conclusions

Immersing different types of resin composites (conventional, “alkasite”, and bulk-fill) and a restorative glass hybrid cement in a green smoothie drink led to softening, roughening, and discoloration of the material’s surface. These results indicate that the acidic pH of the green smoothie drink affects the micromechanical and esthetic properties of restorative materials similarly as previously reported for other acidic drinks. The effect of the green smoothie drink on surface properties was highly material-specific and individual surface properties (microhardness, roughness, and color change) were affected independently of one another. Hence, all of the tested material types can be expected to undergo degradation of surface properties when exposed to green smoothies, with the extent of degradation varying considerably among the materials and individual properties.

## Figures and Tables

**Table 1:** Composition of tested materials provided by respective manufacturers.

Material (Abbreviation) /Manufacturer/Lot No.	Type	Composition
Tetric EvoCeram (TEC)/ Ivoclar Vivadent, Schaan, Lichtenstein/Z01KN4	Conventional resin composite	Urethanedimethacrylate, Bis-GMA, ytterbium trifluoride, ethoxylated bisphenol A dimethacrylate, barium glass filler, ytterbium trifluoride, mixed oxide
Cention N (CEN)/ Ivoclar Vivadent, Schaan, Lichtenstein/Z00HCG	“Alkasite”(Resin composite with reactive glass fillers)	Powder: barium aluminum silicate glass, ytterbium trifluoride, isofiller, calcium barium aluminum fluorosilicate glass, calcium fluoro silicate glass; Liquid: urethane dimethacrylate, tricyclodecandimethanol dimethacrylate, tetramethyl-xylylene, diurethane dimethacrylate, polyethylene glycol 400, dimethacrylate, ivocerin, hydroxyperoxid
Charisma Classic (CHA)/Kulzer, Hanau, Germany/KA10758	Conventional resin composite	Bis-GMA, TEGDMA; Filler load: 61% by volume (60% inorganic filler by volume and pre-polymerized filler), particle size of 0.005–10 µm, barium aluminum fluoride glass.
Equia Forte HT Fil (EQ/EQC)/ GC, Tokyo, Japan/210201A	Glass hybrid	Powder: fluoroaluminosilicate glass, polyacrylic acid, iron oxide Liquid: polybasic carboxylic acid, water
Filtek One Bulk Fill Restorative (FBF)/ 3M, St. Paul, MN, USA/NF15156	Bulk-fill resin composite	Nonagglomerated/nonaggregated 20 nm silica filler, nonagglomerated/nonaggregated 4 to 11 nm zirconia filler, aggregated zirconia/silica cluster filler, ytterbium trifluoride filler consisting of agglomerate 100 nm particles, ERGP-DMA, diurethane- DMA, 1,12-dodecane-DMA

Note: Bis-GMA: bisphenol A-glycidyl methacrylate; PMMA: poly (methyl methacrylate); TEGDMA: triethylene glycol di methacrylate; DMA: dimethacrylate; UDMA: urethane dimethacrylate.

## Data Availability

Not applicable.
